# The brighter side of climate change: How local oceanography amplified a lobster boom in the Gulf of Maine

**DOI:** 10.1111/gcb.14778

**Published:** 2019-08-28

**Authors:** Andrew G. Goode, Damian C. Brady, Robert S. Steneck, Richard A. Wahle

**Affiliations:** ^1^ School of Marine Sciences University of Maine Orono ME USA

**Keywords:** American lobster, biological thresholds, climate change, ocean warming, regional oceanography, thermal habitat

## Abstract

Ocean warming can drive poleward shifts of commercially important species with potentially significant economic impacts. Nowhere are those impacts greater than in the Gulf of Maine where North America's most valuable marine species, the American lobster (*Homarus americanus* Milne Edwards), has thrived for decades. However, there are growing concerns that regional maritime economies will suffer as monitored shallow water young‐of‐year lobsters decline and landings shift to the northeast. We examine how the interplay of ocean warming, tidal mixing, and larval behavior results in a brighter side of climate change. Since the 1980s lobster stocks have increased fivefold. We suggest that this increase resulted from a complex interplay between lobster larvae settlement behavior, climate change, and local oceanographic conditions. Specifically, postlarval sounding behavior is confined to a thermal envelope above 12°C and below 20°C. Summer thermally stratified surface waters in southwestern regions have historically been well within the settlement thermal envelope. Although surface layers are warming fastest in this region, the steep depth‐wise temperature gradient caused thermally suitable areas for larval settlement to expand only modestly. This contrasts with the northeast where strong tidal mixing prevents thermal stratification and recent ocean warming has made an expansive area of seabed more favorable for larval settlement. Recent declines in lobster settlement densities observed at shallow monitoring sites correlate with the expanded area of thermally suitable habitat associated with warmer summers. This leads us to hypothesize that the expanded area of suitable habitat may help explain strong lobster population increases in this region over the last decade and offset potential future declines. It also suggests that the fate of fisheries in a changing climate requires understanding local interaction between life stage‐specific biological thresholds and finer scale oceanographic processes.

## Graphical abstract

Ocean warming has rapidly expanded the amount of thermally suitable bottom habitat for settling American lobster postlarvae in the Gulf of Maine. Local oceanography, coupled with nonlinear responses to warming, may have led to a demographic expansion disproportionate to the relatively small change in bottom temperature. Habitat expansion offshore may explain population increases northeastward and decreases in inshore settlement densities.

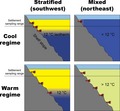

## 1 INTRODUCTION

Global climate change is an information dense, but deceptively complicated, phrase. Most of the world's oceans are warming, but patterns in the distribution, abundance, and rate of warming varies in space, time, and in ways that can have profound effects on marine organisms (Kleisner et al., 2016; Pinsky, Worm, Fogarty, Sarmiento, & Levin, 2013). Several studies have recorded the "climate velocity" of the net poleward shifts in the distribution of marine organisms that are commercially fished (Nye, Link, Hare, & Overholtz, 2009; Pinsky et al., 2013) or ecologically important to marine ecosystems (such as coral reef fish: Vergés et al., 2014; and kelp forests: Wernberg et al., 2010). However, to assess the effects of ocean warming requires understanding how those aspects of climate change affect organisms where they live. Most studies use trends in sea surface temperature (SST) as a convenient and important measure of local warming (e.g., Pershing et al., 2015), but such remote sensing metrics may not be indicative of exposure for organisms with key life history processes occurring on or near the seabed. Such is the case of the northwest Atlantic coastal and shelf waters where thermal properties of sea temperature vary dramatically in time and space (Kavanaugh, Rheuban, Luis, & Doney, 2017).

We focus on the American lobster (*Homarus americanus* Milne Edwards) because it is the most valuable single‐species fishery in North America, with a combined value of more than $US 1.67 billion in 2016 (DFO, 2018; NMFS, 2018), and it is also one of the best studied organisms in the world. Almost a century ago, Huntsman (1923) surmised that despite thriving lobster fisheries, the absence of juvenile lobster in the Bay of Fundy and northeastern Gulf of Maine could be attributed to the colder waters preventing larval development and settlement. Studies over the last three decades determined that the demography of this species is driven by the successful settlement of its pelagic postlarval stage on the seabed (Barshaw & Bryant‐Rich, 1988; Boudreau, Bourget, & Simard, 1990; Boudreau, Simard, & Bourget, 1991; Cobb, Gulbransen, Phillips, Wang, & Syslo, 1983; Incze & Wahle, 1991; Palma, Steneck, & Wilson, 1999; Steneck & Wilson, 2001; Wahle & Incze, 1997; Wahle & Steneck, 1991). More recently, field observations by Annis (2005) determined that postlarvae sounding behavior is restricted to temperatures warmer than 12°C (i.e., the "thermal threshold" sensu Annis, 2005), an observation consistent with subsequent deep‐water settlement surveys (Wahle, Brown, & Hovel, 2013).

Thermal thresholds act in ways that directly impact species fitness and demographic processes. The 12°C threshold for lobster indicates a point at which colder temperatures induce lethal (e.g., irregular respiration and heartbeat; Quinn, 2017) and sublethal (increased time spent in the plankton: MacKenzie, 1988; behavioral avoidance and decreased size at molt: Annis, Wilson, Russell, & Yund, 2013) effects. Small temperature changes about this threshold correspond with large changes in survival and settlement which decouples larval supply from settlement patterns (Annis et al., 2013). Thus, the 12°C threshold can act as an ecological barrier to larval transport (Tilburg, McCartney, & Yund, 2012) and survival. Ocean warming, therefore, has the potential to significantly impact the distribution and survival of lobster larvae. This is especially the case in the Gulf of Maine where end‐of‐century projections suggest an expanded area and time the seabed spends above the thermal threshold (Rheuban, Kavanaugh, & Doney, 2017).

Here, we examine the interrelationships of seabed temperatures along the oceanographically distinct Gulf of Maine and how they relate to lobster demography after larval settlement. Specifically, we suggest that the combination of thermally mediated sounding behavior of lobsters, particularly in areas susceptible to ocean stratification, decouples the easily measured SST from the more relevant bottom temperatures. This not only paints a different picture of how ocean warming may affect the distribution and abundance of the American lobster but it also alters how we interpret decades of young‐of‐year (YoY) settlement data that have consistently declined for approximately a decade in the Gulf of Maine. At stake is whether the single most valuable fisheries species in North America is on a trajectory of decline.

## 2 METHODS

### 2.1 Study area

**Figure 1:**
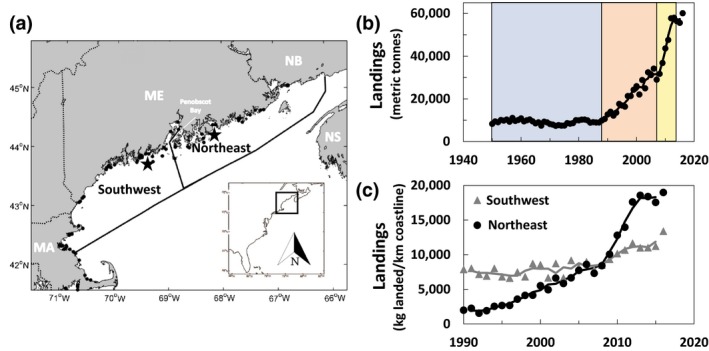
(a) Southwest and northeast and Gulf of Maine. Black dots: survey locations of the American lobster settlement index. Black stars: NERACOOS buoy locations. Dotted lines: state or country political boarders; ME: Maine, MA: Massachusetts, NB: New Brunswick, NS: Nova Scotia. Inset: East coast of the United States. (b) Phases of Maine's lobster fishery 1950–2017: Stasis (1950–1988), linear growth (1989–2008), geometric growth (2009–2013). (c) Southwest–northeast differences of the Gulf of Maine lobster landings per length of coastline during periods of growth (1990–2016). Since 1990, landings in the southwestern Gulf of Maine have increased gradually, but have increased rapidly in the northeast, especially since 2008. Solid lines denote 3 year moving block average

The Gulf of Maine (Figure 1a) is a semienclosed continental shelf sea that is bounded by Cape Cod, Massachusetts, USA, and Nova Scotia, Canada (Figure 1a). It has supported some of the most productive fisheries in the world; most notably groundfish (e.g., Atlantic cod; Alexander et al., 2009), ocean scallop (MDMR, 2018; NMFS, 2018), and lobster (DFO, 2018; MDMR, 2018; NMFS, 2018). Currently, the American lobster supports the most valuable fishery in the United States and Canada, and 90% of the US production comes from the Gulf of Maine (NMFS, 2018). The region's high biological productivity (Bigelow, 1926; O'Reilly & Busch, 1984) derives from nutrient rich, deep slope water (Townsend, Thomas, Mayer, Thomas, & Quinlan, 2006) and Scotian Shelf water (Townsend, 1997, 1998) flowing into the Gulf of Maine near Nova Scotia. After these waters enter the Gulf of Maine, the Eastern Maine Coastal Current is generated by cyclonic baroclinic circulation directing water toward the Bay of Fundy (Brooks, 1985; between New Brunswick and Nova Scotia), intense mixing due to some of the world's largest and strongest tides (Townsend et al., 2006), flow along the coast of eastern Maine, and an offshore plume just prior to Penobscot Bay (Bisagni, Gifford, & Ruhsam, 1996; Brooks & Townsend, 1989; Pettigrew et al., 1998; Townsend, Christensen, Stevenson, Graham, & Chenoweth, 1987). Southwestward of Penobscot Bay, the Western Maine Coastal Current is generated by freshwater discharge from rivers aided by winds and cyclonic baroclinic circulation (Bigelow, 1927; Brooks, 1985; Fong, Geyer, & Signell, 1997; Franks & Anderson, 1992). These two current systems act to divide the coast of the Gulf of Maine into northeastern and southwestern sectors, each having unique physical characteristics; colder and well mixed to the northeast, warmer and stratified to the southwest.

### 2.2 Lobster landings

The State of Maine lobster fishery has one of the longest time series of lobster landings in the world dating back to 1880 (MDMR, 2018). To the extent that landings (integrated over several years) are a reliable predictor of lobster population size (Steneck & Wilson, 2001), these historical data help us identify population trends over the past century. Maine landings were used to identify the time frame over which landings have changed. To compare landing trends from areas with very different coastline lengths along the coast of the Gulf of Maine, landings over this time frame were standardized per unit length of coastline (e.g., Steneck & Wilson, 2001). Specifically, we incorporated landing data from Canadian lobster fishing area 36 (DFO, 2018), Maine state lobster management zones (MDMR, 2018), New Hampshire (NMFS, 2018), and Massachusetts Division of Marine Fisheries zones 1–5 (T. Pugh – Massachusetts Division of Marine Fisheries, personal communication, 2018). Coastline length was determined by ArcGIS.

### 2.3 Characterizing thermal habitat

Many recent studies necessarily use SST to characterize temperature exposure of benthic organisms. In this study, we used numerical model output that assimilates SST but has the advantage of using local bathymetry and explicitly incorporates tidal mixing to improve bottom water temperature estimates. NOAA's Northeast Coastal Ocean Forecasting System (NECOFS; an implementation of the Finite Volume Community Ocean Model [FVCOM]) was used to estimate year‐to‐year variability in the area of a thermally suitable seabed for lobster settlement. NECOFS is a three‐dimensional ocean circulation model codeveloped by the University of Massachusetts‐Dartmouth and the Woods Hole Oceanographic Institution (Chen, Beardsley, & Cowles, 2006). NECOFS is well suited to simulate geophysical marine environments characterized by complex coastlines due to its flexibility in mass conservation, triangular grid geometric flexibility, and computational efficiency (Chen et al., 2006). In this study, the NECOFS FVCOM‐G3 grid was employed. The unstructured NECOFS FVCOM‐G3 grid provides a horizontal resolution ranging between ~20 m inshore and ~10 km at the model open‐boundary (Chen et al., 2006). Hourly bottom temperature data are modeled at 48,451 nodes and demonstrate a high reliability to predict bottom water temperatures (Li, Tanaka, Chen, Brady, & Thomas, 2017).

Daily bottom temperature output was collected during the lobster settlement season (August–October) from 1978 to 2015. Daily bottom temperatures were averaged over the lobster settlement season (August–October) per grid node per year. Average grid temperatures were calculated as the average seasonal temperature of the grid's nodes. The area of bottom habitat per grid were determined by using node depth via the USGS 15‐arcsec digital bathymetry dataset (Roworth & Signell, 1998) and using Heron's formula:

$$\text{Area}=\sqrt{s(s-a)(s-b)(s-c)},$$

$$s=\frac{a+b+c}{2},$$

where *a*, *b*, and *c* are the side lengths, and *s* is the semiperimeter of each triangular grid. Average bottom temperatures of all nodes with average depths between 0–25, 25–50, and 50–100 m were calculated per year. The least‐squares regressions were determined per node and a one‐way ANOVA was used to determine significant differences in warming rate per depth stratum. The annual bottom area of suitable thermal habitat (≥12°C; Annis, 2005) were determined for the northeastern and southwestern Gulf of Maine.

We also used data from Northeastern Regional Association of Coastal Ocean Observing Systems (NERACOOS) buoys to evaluate the relationship between observed surface and bottom temperatures in the northeastern and southwestern Gulf of Maine. As a component of NERACOOS, the University of Maine Physical Oceanography Group develops, operates, and manages the Gulf of Maine buoy array that was founded in 1999 as the Gulf of Maine Ocean Observing System. Data were collected from two buoys that are approximately equidistant from the mouth of Penobscot Bay (I01: Northeast of Penobscot Bay, LAT: 44.10, LON: −68.10 and E01: Southwest of Penobscot Bay, LAT: 43.71, LON: −69.35). These locations were chosen because they represent the stratification differences between the two regions in the Gulf of Maine. We compared daily average surface (1 m) and bottom (50 m) water temperatures over the lobster settlement season (August–October) from 2001 to 2017. The least‐squares regression of surface‐to‐bottom temperatures and a two‐tailed *t* test assuming unequal variances on the mean surface‐to‐bottom temperature differences were performed to determine water column thermal structure and the magnitude of the southwest‐to‐northeast difference. We also compared daily surface (1 m) and bottom (50 m) buoy temperatures to modeled NECOFS temperatures at the nearest corresponding sigma layer depth by least‐squares regressions from 2001 to 2015.

### 2.4 The American Lobster Settlement Index

The American Lobster Settlement Index (ALSI) is an annual monitoring program that quantitates settlement densities of YoY lobster across New England and Atlantic Canada. ALSI was initiated in 1989 at a few sampling locations in midcoast Maine (Incze & Wahle, 1991) and has since expanded to more than 100 sites ranging from Rhode Island, USA, to Newfoundland, CAN sampled either by diver‐based suction sampling (Incze & Wahle, 1991; Wahle & Steneck, 1991) or by passive postlarval collectors (Wahle, Bergeron, et al., 2013; Wahle, Bergeron, Wilson, & Parkhurst, 2009). The Gulf of Maine time series used in this study was entirely generated by the diver‐based method. Sampling is conducted at the end of the settlement season in September and October. YoY lobster densities are an indicator of lobster year class strength (Incze & Wahle, 1991; Incze, Wahle, & Cobb, 1997; Wahle & Incze, 1997) and have been used to predict trends in fishery recruitment (Oppenheim, 2016; Wahle, Gibson, & Fogarty, 2009).

Annual YoY settlement densities were averaged over all sampling sites for the northeastern and southwestern Gulf of Maine. To minimize bias introduced by the increasing number of sites sampled as ALSI expanded its geographic range, YoY settlement data in this study were only used from years (2000–2015) when the number of sampling sites exceeded half the maximum number of sampling sites per region in the Gulf of Maine (17 in the northeastern Gulf of Maine; 41 in the southwestern Gulf of Maine; Figure S1).

### 2.5 Thermal habitat and YoY settlement

We reasoned that if the area of thermally suitable seabed varied in time, interannual variability in YoY density alone could misrepresent the true time trend in lobster year class strength. One consequence of expanding habitat could be that larval settlement spreads over a larger area with correspondingly reduced densities while extrapolated abundances may be stable or even increasing. We refer to this as the “thermally mediated dilution hypothesis.” As a partial test of that hypothesis, we evaluated the relationship between the area of seabed warmer than 12°C (from NECOFS) and YoY lobster density. For each region we conducted least‐squares regressions between NERACOOS SST versus YoY density, NERACOOS bottom temperature versus YoY density, NECOFS SST versus YoY density, NECOFS bottom temperature versus YoY density, and seabed area >12°C versus YoY density using annual values and 3 year moving block averages from 2000 to 2015. Three year moving block average values were used in an attempt to minimize the influence of other elements on inshore lobster settlement patterns, including abiotic and biotic factors modulating larval supply (Xue, Incze, Xu, Wolff, & Pettigrew, 2008), copepod (Carloni, Wahle, Geoghegan, & Bjorkstedt, 2018) and demersal fish abundance (Wahle, Bergeron, et al., 2013) effects on natural mortality of pre‐ and postlarvae, spatiotemporal variability of competent larvae and suitable thermal habitat, proximity to land boundaries (Steneck & Wilson, 2001), sediment availability and use, and fixed station sampling that may underrepresent annual lobster recruitment (Li, Cao, Chang, Wilson, & Chen, 2015).

We estimated the depth over which lobster settlement is relatively homogenous by reanalyzing data from Wahle, Bergeron, et al. (2013). Our analysis focused on data from the northeastern Gulf of Maine where temperature was uniform across depths and was unlikely to influence depth‐wise patterns of settlement. We standardized annual settlement densities over the three depth strata (0–25, 25–50, and 50–100 m) to shallow settlement densities over the study period (2007–2008). We then performed a one‐way ANOVA and Tukey–Kramer post hoc test to determine the depth at which settlement density significantly deviated from shallow settlement densities. Based on this analysis, we chose 50 m as the limit for the relatively uniform distribution of YoY lobster over suitable thermal habitats (Figure S2).

### 2.6 Extrapolating YoY settlement over thermally suitable habitats

On the strength of the inverse relationship between YoY density and area of thermally suitable habitat, we generated two additional indices of YoY abundance, which extrapolate YoY density over areas of a thermally suitable seabed in different ways. The first assumes uniform settlement across depths shallower than 50 m (Wahle, Bergeron, et al., 2013; see above). In this case, extrapolated recruitment, *R*, was taken as the product of YoY settlement density, *ρ* , from shallow diver‐sampled locations and the area, *A*, of thermally suitable sea bed subject to temperatures >12°C:

R=ρ∗A.

The second scales YoY settlement density across three depth strata (0–25, 25–50, and 50–100 m) and further adjusts it for the availability of suitable geological substrate:

$$R^{\prime }=\sum_{z=1}^{3}\left(D_{Z}*H_{Z}*\rho *A_{Z}\right),$$

where *R′* is the extrapolated YoY recruitment, *Z* is depth strata (1 = shallow: 0–25 m, 2 = mid: 25–50 m, and 3 = deep: 50–100 m), *D* is the depth scaling factor, *H* is the habitat scaling factor, *ρ* is inshore YoY lobster density, and *A* is the bottom area of thermal habitat >12°C. We determined the depth scaling factor using a least‐squares regression of deep‐water settlement data taken from Wahle, Bergeron, et al. (2013); see above (Figure S2). On the basis of prior research, we assumed rocky substrate to be the most suitable shelter‐providing settlement habitat and therefore further scaled our YoY recruitment estimate according to the availability of that habitat. Toward this end we linearly interpolated the coverage of rocky habitat from the United States. Geological Survey east‐coast surficial geology database (USGS, 2005) onto the NECOFS grid (Figure S3). Since relative settlement success is not known for alternative habitat types (Chassé & Miller, 2010), we assumed binary recruitment success: whereby the proportion recruited onto rocky habitat is 1 and nonrock habitat is 0.

## 3 RESULTS

Lobster landings increased over the past 30 years which lead the Gulf of Maine lobster fishery to attain its record status in the United States as the most valuable single‐species fishery (NMFS, 2018; Figure 1). Maine's historical landings trend since 1950 has three distinct phases (Figure 1b). First, protracted stasis from 1950 to the 1980s with landings around 10,000 MT. Then a linear increase in landings until about 2008; after which landings increased geometrically to about 60,000 MT (Figure 1b).

However, the northeast and southwest sectors (Figure 1a) showed distinctively different trajectories in landings on a per unit coastline basis (Figure 1c). In the early 1990s, the southwest region landed four times as many lobsters per unit length coastline as the northeast. At that time, landings in both entered a linear growth phase (~1989–2008). Northeastern landings grew much faster than southwestern landings during this phase so that by ~2007 landings per unit coastline were similar in northeastern and southwestern Maine. Soon thereafter, the northeastern coastal fishery entered a phase of geometric growth, greatly surpassing landings in the southwest even as they continued to grow, so that by 2015 the northeastern landings per unit coastline were 80% higher than in the southwest (Figure 1c).

**Figure 2:**
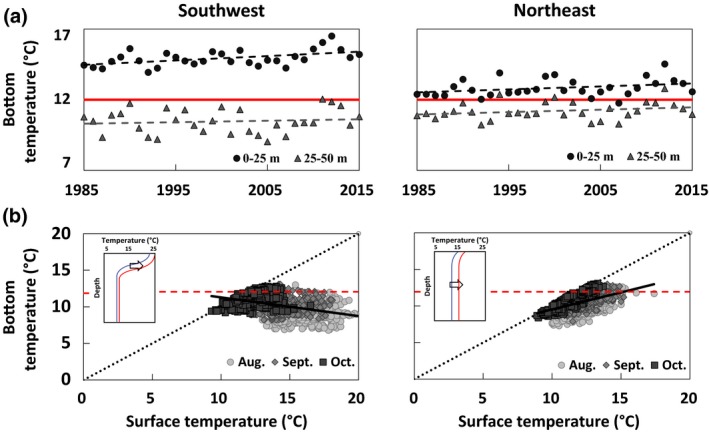
Two ways of viewing southwest–northeast differences in the Gulf of Maine's thermal regime and warming. (a) FVCOM‐modeled Aug. to Oct. bottom temperatures 1985–2015 and rates of change in two depth zones. Solid red line: 12°C threshold. Dashed lines: least‐squares regressions. Regression statistics can be found in Table S3. (b) Relationship between observed surface (1 m) and bottom (50 m) temperatures at NERACOOS buoys in southwestern and northeastern Gulf of Maine, 2001–2017. Southwest: *Y* = −0.256*x* + 13.9, *N* = 1,524, *R* ^2^ = 0.18, *p* < .001. Northeast: *Y* = 0.467*x* + 4.9, *N* = 1,556, *R* ^2^ = 0.22, *p* < .001. Black dotted line: 1:1 relationship; when points fall on that line, the water column is well mixed. Red dashed line: 12°C threshold. Inset: Interpretation of how east and west summer temperature profiles change in a warming ocean (black arrow)

Northeast–southwest differences in thermal regime, water column structure, and ocean warming are largely driven by differences in the degree of vertical mixing (Figure 2). Summer maxima in the northeastern Gulf of Maine typically ranges from 11 to 13°C uniformly across all depths to 100 m, whereas in the southwestern Gulf of Maine summer temperatures may reach 16–18°C at the surface, but only 7–8°C at depths >50 m. Average summer temperatures from 0 to 25 m have exceeded the 12°C lobster settlement threshold in both regions; however, warming trends suggest that this has occurred only recently (~1980s) in the northeastern Gulf of Maine. In contrast, temperatures between 25 and 50 m in the northeast were considerably warmer than the southwest and reached the 12°C settlement threshold starting in 1999–2000. At depths between 25 and 50 m, temperatures have warmed at similar rates between the southwest and northeast. However, bottom temperatures have warmed considerably faster between 50 and 100 m in the northeast causing the seabed to approach the 12°C settlement threshold sooner than the southwest (*F*_5, 6,981_ = 32.29, *p* < .001; Table S3). Modeled surface‐to‐bottom temperature differences are further corroborated by in situ buoy measurements that are significantly linearly related (Figure S4). Water column temperature differences are significantly higher in the southwest (*M* = 4.1, *SD* = 3.0) compared to the northeast (*M* = 1.6, *SD* = 1.1); *t*_20,20_ = 31.1, *p* < .001. Modeled and in situ data both show that strong summer stratification in the southwestern Gulf of Maine restricts warming to the upper water column causing bottom waters to remain cold (Figure 2b) and warm at a slower rate (Figure 2a). In contrast, the northeastern Gulf of Maine is well mixed causing surface and bottom water temperatures to converge (Figure 2b) and warm at similar rates (Figure 2a).

**Figure 3:**
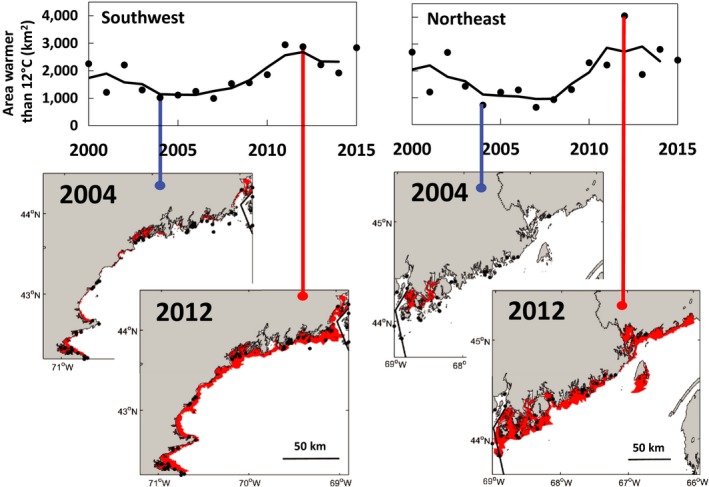
Estimated bottom habitat with temperatures >12°C (km^2^) shallower than 50 m in the southwestern and northeastern Gulf of Maine between 2000 and 2015 from NECOFS modeled bottom temperatures. Inset maps compare the area of seabed subject to temperatures >12°C during a cool year (2004) and a warm year (2012) in the two areas. Black dots on maps are American lobster settlement index sampling locations. Solid lines denote a 3 year moving block average

**Figure 4:**
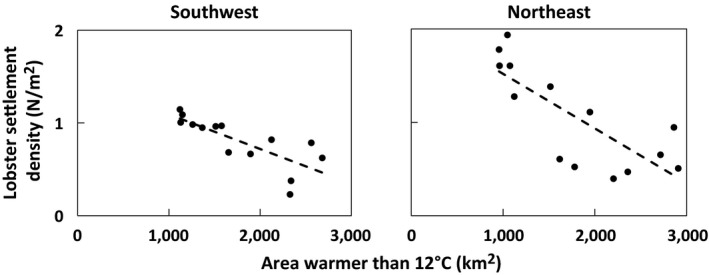
Relationship between thermal habitat area (km^2^) and young‐of‐year lobster densities (N/m^2^) in the southwestern and northeastern Gulf of Maine. Southwestern Gulf of Maine: *Y* = −3.74 × 10^−4^ *x* + 1.47, *N* = 14, *R* ^2^ = 0.60, *p* = .001. Northeastern Gulf of Maine: *Y* = −5.86 × 10^−4^ *x* + 2.11, *N* = 14, *R* ^2^ = 0.61, *p* = .001. Three‐year moving block average values were used

The area of seabed exposed to temperatures warmer than 12°C has changed over the past decade and a half. In both regions, this area declined from 2000 to 2007, increased from 2007 to 2012, and declined again from 2012 to 2015 (Figure 3). The range of habitat area warmer than 12°C is greatest in the northeast compared to the southwest. However, the magnitude of this difference depends on the depth to which thermal habitats are considered (Figure S5). The northeast has significantly higher variances, at the 0.05 level, of the area of seabed warmer than 12°C from 2000 to 2015 for regions shallower than 25 m (*F*_15,15_ = 2.66, *p* = .03) and 100 m (*F*_15,15_ = 3.64, *p* = .01), but is marginally insignificant for regions shallower than 50 m (*F*_15,15_ = 2.25, *p* = .06). In the southwestern Gulf of Maine, because of the steep thermocline, habitat area dynamics were limited to a limited range of depths. In the northeastern Gulf of Maine, vertical mixing and increased susceptibility in offshore boundary heat flux dynamics (Mountain, Strout, & Beardsley, 1996) increased the suitable thermal habitat resulting in both a northeastward and depth‐wise expansion. In both regions of the Gulf of Maine, we found significant negative correlations between the area of seabed warmer than 12°C and YoY density (Figure 4; southwestern Gulf of Maine: *R* ^2^ = 0.60, *p* = .001, *n* = 14; northeastern Gulf of Maine: *R* ^2^ = 0.61, *p* = .001, *n* = 14) indicating that periods of high habitat availability correlate with periods of low settlement density. The slopes of these relationships were not statistically different between regions; *t*(24) = 1.31, *p* = .20. The strength of the inverse relationship between the area of seabed warmer than 12°C and YoY density was relatively constant when regressions were performed on thermal habitats 0–25, 0–50, and 0–100 m (Table S1). Similar inverse relationships between surface and bottom temperatures to settlement density were found, indicating that periods of warmer ocean temperatures correspond with periods of low settlement density (Table S2).

**Figure 5:**
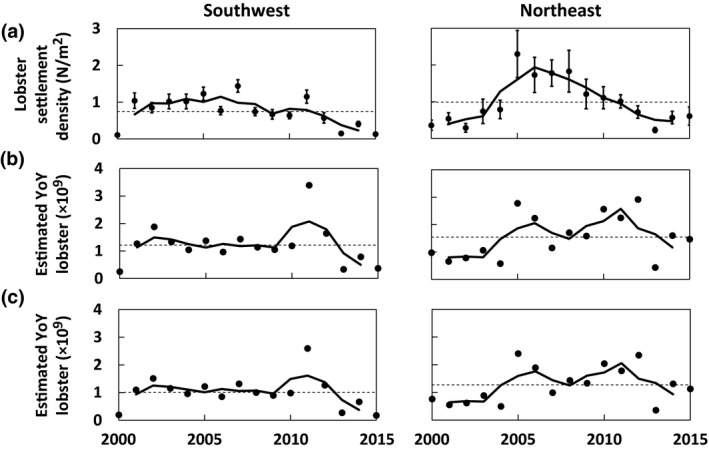
Implications of expanded habitat for lobster settlement. (a) Reported mean densities of young‐of‐year (YoY) lobster ≤10 m from 2000 to 2015 in the southwestern and northeastern Gulf of Maine. (b) Extrapolated abundance of YoY lobster uniformly distributed over suitable thermal habitats ≤50 m. (c) Extrapolated abundance of YoY lobster over suitable thermal habitats ≤100 m taking into account depth‐dependent scaling of inshore (≤10 m) YoY densities and rocky habitat availability. Solid line denotes the 3 year moving block average. Dashed line denotes time‐series average

The YoY lobster time series with and without expanded habitat give alternative views of settlement trends, and thus have important implications for year class strength (Figure 5). We start in 2000, the year greater than 50% of the current monitoring sites are represented. In the southwestern Gulf of Maine, unadjusted YoY densities rose slightly to a high in ~2005–2007 before declining about fourfold to a time‐series low in 2015 (Figure 5a). Incorporating expanded thermal habitat offsets those declines and implies that YoY abundance has remained relatively stable in the southwestern Gulf of Maine through 2010, rose sharply in 2011, and then fell to time‐series lows after 2013 (Figure 5b,c). Both extrapolated YoY abundances follow this trend, but the magnitude of the 2011 peak and time‐series average are 30% and 20% lower, respectively, in the depth and substrate scaled model. Attention should also be given to the 2011 peak as it falls outside 3x the interquartile range of the time series for both models, typically characteristic of an extreme outlier (Tukey, 1977); however, similar extreme recruitment events are common in marine populations (e.g., Hjort, 1914). In the northeastern Gulf of Maine, YoY densities rose sharply from 2000 to sustained high levels ~2005–2008, roughly twice the peak levels in the southwestern Gulf of Maine. Subsequently, northeastern ALSI declined sharply 10‐fold to a time‐series low in 2013 (Figure 5a). Incorporating thermal habitat expansion generates two peaks in abundance, one near 2005 and another in 2012, followed by a decline to near the time‐series average by 2015 (Figure 5b,c). Both extrapolated YoY abundances follow this trend; however, the depth and substrate scaled model produces a 21% lower time‐series average, 24% lower 2012 peak, 16% lower 2005 peak, and a maximum value occurring in 2005 rather than in 2012. In effect, extrapolating densities over the area of thermally suitable habitat advances time‐series highs, and in the northeastern Gulf of Maine it reduces the magnitude of potential decline from 10‐ to twofold.

## 4 DISCUSSION

We illustrated that lobster landings have been increasing for decades with the greatest expansion in northeastern Maine (Figure 1c). This is consistent with studies of climate velocities attributed to rising ocean temperatures (Pinsky et al., 2013) and contrasting rates of landings increase in the Gulf of Maine. However, often, the mechanisms driving these changes in distribution are unknown or difficult to quantitate. SST, used in most studies of climate–fisheries interactions (e.g., Pershing et al., 2015), offer generalized trends in ocean conditions. It is only when we couple these trends to biological thresholds that we get closer to process‐level understanding and improve our predictive capability. We considered warming in the context of thermally mediated lobster settlement behavior (Annis, 2005). It is influenced by the strong tidal mixing in the northeast region of the Gulf of Maine (Figure 2), and we offer a mechanistic explanation for an economically important species that has been shown to have settlement‐driven demography (e.g., Incze et al., 1997; Palma et al., 1999; Wahle, Bergeron, et al., 2013; Wahle & Incze, 1997).

We demonstrated that the impacts of climate change are spatially variable in the Gulf of Maine due to local oceanographic conditions that change the susceptibility of nearshore areas to warming. Northeastern Gulf of Maine warming is largely uncoupled to synoptic conditions due to strong tidal mixing (Mountain et al., 1996), and is most likely driven by offshore advective components of heat flux. However, interannual variability in surface heat flux in southwestern Gulf of Maine is highly correlated with local heat flux due to the importance of stratification in this region (Figure 2). These local oceanographic conditions have resulted in uneven warming rates across the Gulf of Maine and the degree to which warming influences the seabed (Kavanaugh et al., 2017; Figure 2). As a consequence, thermogeographic patterns of YoY lobster recruitment have followed the patterns of benthic warming.

Recent seabed warming in the northeastern Gulf of Maine has surpassed the lower physiological threshold for lobster settlement and rapidly expanded the suitable thermal habitat for lobster northeastward (Figure 3). By virtue of a well‐mixed water column, warming has made a larger range of depths more suitable for juvenile lobster in the northeastern compared to the southwestern Gulf of Maine. We suggest that the expanded thermal habitat may have played a significant role in the unprecedented surge in landings in the northeastern Gulf of Maine and could explain the decline in settlement density at shallow water monitoring stations as the area of the thermally suitable seabed expanded (Figure 4). Thus, cooler than average years (e.g., 2004) and warmer than average years (e.g., 2012) show a waxing and waning of potential nursery habitat area, respectively (Figure 3). Over the past decade as waters warmed, settlement densities in shallow areas have declined (Table S2), but these declines may have been offset by expanded thermal habitats (Figure 3) and increased depth of larval sounding behavior (Annis, 2005). To the extent that trends in recruitment predict trends in landings, these results suggest an alternate future for the American lobster fishery, which has been predicted to be on the verge of an imminent decline due to the decline in the density of observed settlers (Le Bris et al., 2018; Oppenheim, 2016). Our two methods of estimating newly settled lobster abundance suggest relative stasis (or a slight increase) in the southwestern Gulf of Maine and a somewhat more positive outlook for near‐term landings in the northeastern Gulf of Maine than would be suggested simply from shallow settlement densities.

The YoY recruitment time series with and without expanded habitat give alternative views of settlement trends, and thus have important implications for year class strength and stock assessment. To the extent settlement trends may predict subsequent fishery recruitment (e.g., Oppenheim, 2016; Wahle, Bergeron, et al., 2009), the unmodified ALSI YoY index suggests an impending downturn, whereas the index extrapolated over thermally suitable habitat suggests a more sustained trajectory of high abundance and a delayed downturn. Currently, the ALSI YoY densities are included in the American lobster stock assessment as an indicator of the health of the resource (ASMFC, 2015); however, environmental variability, such as thermally suitable habitat, is not included. Similar to our findings, incorporating environmental variability into the lobster stock assessment model impacts estimates of annual recruitment (Tanaka et al., 2019). Thus, it may be especially important to account for environmental variability and rapid demographic changes across critical biological thresholds, such as that observed in the northeastern Gulf of Maine: roughly a doubling in optimal thermal habitat from 2008 to 2012 (Figure 3). Consequently, YoY density corrections incorporating environmental variability may provide a more accurate representation of lobster recruitment in a rapidly warming part of the ocean.

Deep‐water settlement data obtained by the ALSI research collaborative along New England and Atlantic Canada from 2007 to 2008 (Wahle, Bergeron, et al., 2013) and 2016 to 2018 (R. Wahle, unpublished) provide corroborating evidence of depth‐wise patterns of settlement. They confirm how strongly depth‐wise settlement patterns in the Gulf of Maine are influenced by the degree of thermal stratification. In the northeast GoM, settlement below 25 m has been on the order of nine times as high as at the same depth in the southwest. It is, therefore, more likely that the boom in landings in the east was amplified by a broader depth‐wise component not present in the western GoM. Regrettably, no deep‐water settlement data are available prior to 2007 that might have better documented the status of settlement patterns in the preboom years.

These results, however, fail to directly support or refute the thermally mediated dilution hypothesis. Observations from ALSI deep‐water settlement collectors, ventless trap surveys, and inshore trawl surveys do not yet provide a direct test of the thermally mediated dilution hypothesis. Although Maine lobster landings have shown signs of leveling off since 2013, consistent with the onset of declines in shallow water settlement density after 2008, ongoing fishery independent surveys, such as ventless trap surveys and inshore trawl surveys have failed to reveal declines in sublegal lobsters that might have been expected to lead the landings decline. These surveys appear to corroborate an increase in abundance across life stages at depths exceeding 25 and 50 m (Sherman, Stepanek, Pierce, Tetrault, & O'Donnell, 2015; Figure S6). However, these methods may introduce their own biases related to the interaction between warming waters and lobster catchability causing annual patterns to potentially misrepresent recruitment strength. Continued multilife stage monitoring over a full range of depths is, therefore, necessary to confirm how settlement and movements of postsettlement stages relate to water column thermal properties.

The thermally mediated dilution hypothesis is not the only potential explanation of declining recruitment density. Declining reproductive output (Koopman, Westgate, & Siders, 2015), recruit‐per‐egg ratios (Le Bris et al., 2018), and lipid‐rich prey of larvae (Carloni et al., 2018) have been proposed as mechanisms driving declines in recruitment density under warming conditions. Consequently, Le Bris et al. (2018) project that recruitment in a warming Gulf of Maine will show widespread declines across the American lobster's range with only slight increases projected for the northeastern Gulf of Maine. The assumption of reduced recruitment naturally leads to the conclusion that the Gulf of Maine lobster fishery is on the precipice of an imminent decline (Le Bris et al., 2018; Oppenheim, 2016). However, these alternative hypotheses may not fully capture patterns of lobster demography in the Gulf of Maine. Recruitment patterns predicted from SST alone may underestimate recruitment in stratified regions where there are significant surface‐to‐bottom thermal differences. Additionally, declining per capita egg production could be offset by increased spawning stock biomass (ASMFC, 2015; Le Bris et al., 2018; Sherman et al., 2015) facilitated by an increased area of suitable habitat offshore (Tanaka & Chen, 2016). Since the abundance of stage‐one larvae has been shown to be significantly correlated with the abundance of spawning stock biomass (Carloni et al., 2018) it would stand to reason that stage‐one larvae have increased alongside spawning stock biomass. However, annual fluctuations in spawning stock biomass, temperature, spawn timing, and coastal current characteristics may impact the total amount of competent stage‐four larvae transported across suitable recruitment habitats. Finally, the degree to which larval lobsters are food limited in the plankton is not well understood (Carloni et al., 2018). Nonlinear effects of warming on multiple life stages make even short‐term projections of recruitment exceedingly difficult. However, each effort to do so highlights processes that merit observation; in this case, determining whether deep‐water settlement can offset nearshore declines in abundance, and how this may affect landings forecast models.

While seabed warming has had a largely positive demographic effect on lobster in the Gulf of Maine, this has not been true for the southern segment of the population. South of Cape Cod, Massachusetts, USA, entirely different oceanography and thermogeography results in warming temperatures exceeding the 20°C upper threshold and has been associated with physiological stress, rising prevalence of epizootic disease (e.g., Castro & Angell, 2000; Glenn & Pugh, 2006), and with mass mortalities (Pearce & Balcom, 2005). The widespread mortality in southern New England contributed to the reduced reproductive potential and settlement (Wahle, Dellinger, Olszewski, & Jekielek, 2015). Southwestern‐most parts of the Gulf of Maine occasionally exceed 20°C, but these periods are relatively short in comparison to southern New England where regions can exceed this threshold for over 2 months (Wahle, Bergeron, et al., 2013). Northeastern parts of the Gulf of Maine rarely exceed this upper threshold, and the relatively stable thermal regime may buffer this part of the Gulf of Maine from lobster declines related to excessive future warming. Poleward migration of other taxa including predatory fishes will have unknown impacts on American lobsters (Nye et al., 2009; Wahle, Bergeron, et al., 2013). Nevertheless, our understanding of the role of climate change on marine organism distribution requires a better understanding of important details of both the oceanographic and organism attributes.

Clearly, the role of climate change on the distribution and abundance of organisms is steadily gaining attention in all sectors from the public to managers and policy makers. Rates of poleward migration or "climate velocity" of commercially important species have been well documented (Nye et al., 2009; Pinsky & Fogarty, 2012) and generally conform to well‐established patterns of thermogeography driving biogeography (Adey & Steneck, 2001; Hutchins, 1947). However, beyond simple patterns of temperature and organism distribution are complex processes and mechanisms (such as a thermal envelope) driving those patterns. Our proposed mechanistic explanation of climate velocities incorporates warming in the context of realized essential habitat (e.g., nursery habitat) and biological thresholds for a commercially important species. When seabed temperatures exceed the thermal threshold, the probability of lobster settlement onto previously cooler and uninhabitable areas increased. However, contrasting stratified and unstratified water masses differentially affects seabed warming and thus lobster recruitment.

Global models of climate change will continue to provide a valuable coarse‐scale view of the impact of ocean warming on world fisheries, but they do not capture finer scale coastal processes that surely affect how fishery productivity will play out on a local scale. We demonstrate here that local oceanography, coupled with nonlinear species‐specific responses to warming, may have led to a demographic expansion disproportionate to the relatively small change in temperature. By virtue of local differences in vertical mixing, the Gulf of Maine has different susceptibilities to offshore warming that can lead to differences in future productivity. In the case of the American lobster, we argue that ocean warming drove a northeastward population surge in the Gulf resulting from an expansion of the area of seabed across a biologically important thermal threshold for larval settlement. We suggest this northeastward expansion largely contributed to the historic sixfold increase in lobster harvests that has elevated the fishery to its high national ranking in value. We, therefore, argue that the fate of fisheries in a changing climate requires a better understanding of interactions between local oceanography and species traits.
