# Supplementary material for: The brighter side of climate change: How local oceanography amplified a lobster boom in the Gulf of Maine
Source: Glob Chang Biol. 2019 Aug 28;25(11):3906–17. doi: 10.1111/gcb.14778 (PMC6852103; doi:10.1111/gcb.14778)
Supplement: Supplementary file 1 [file GCB-25-3906-s001.docx]

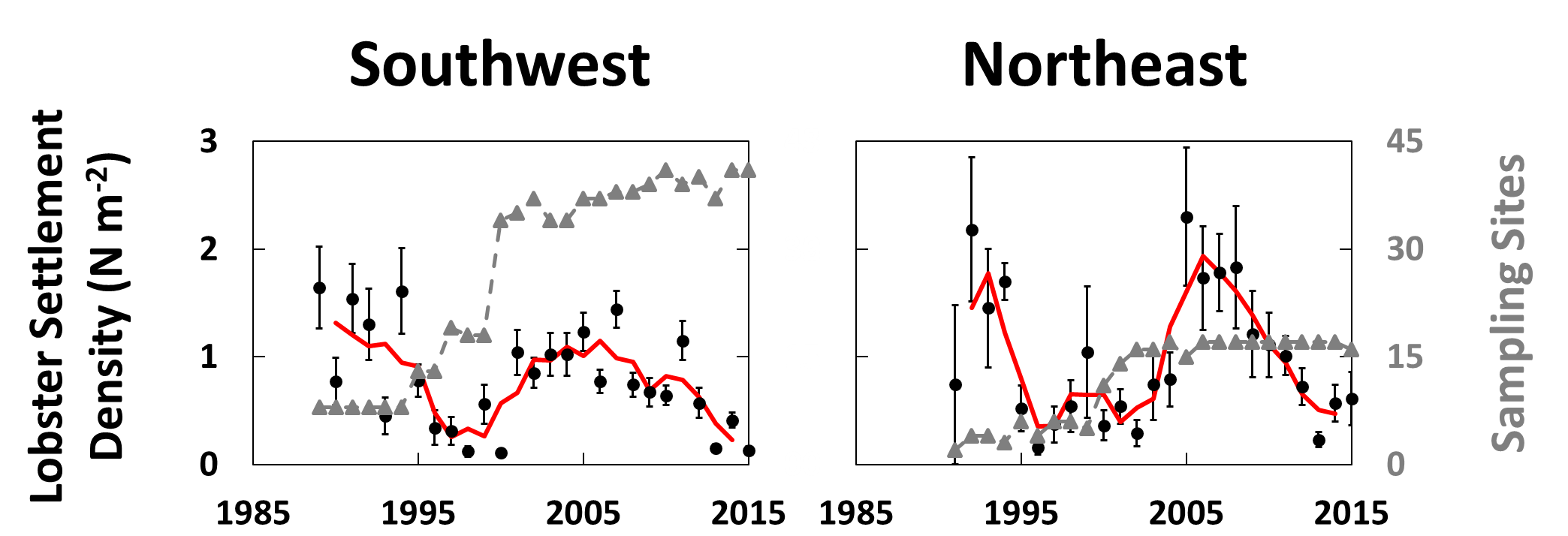


**Supplemental Figure 1**. Progression of the American lobster settlement index (ALSI). Reported mean densities of young-of-year (YoY) lobster < 10 m and number of sites sampled from 1989 and 1991 to 2015 for the southwestern and northeastern Gulf of Maine, respectively. Black circles: YoY settlement density, Grey triangles: number of ALSI sampling sites, Red line: three-year YoY settlement density moving block average.


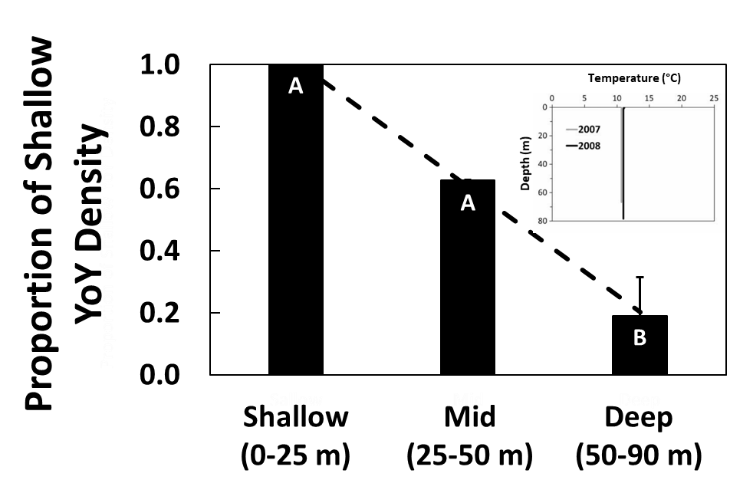


**Supplemental Figure 2**. Combined deep-water settlement patterns from Wahle et al. (2013). Settlement densities were standardized to shallow settlement densities and averaged over the study period (2007-2008) in the northeastern Gulf of Maine. Error bars are standard error. One-way ANOVA summary: F_2, 3_ = 32.29, p = 0.009. A post hoc Tukey HSD test showed significant differences in settlement density between depth strata at the p < 0.05 level. Letters denote statistical similarity. Dashed line: least-squares linear regression; Y = -0.32x + 1.30, N = 6, R^2^ = 0.95, p < 0.001. Inset: temperature profiles where collectors were deployed (Wahle et al. 2013; Figure 5).


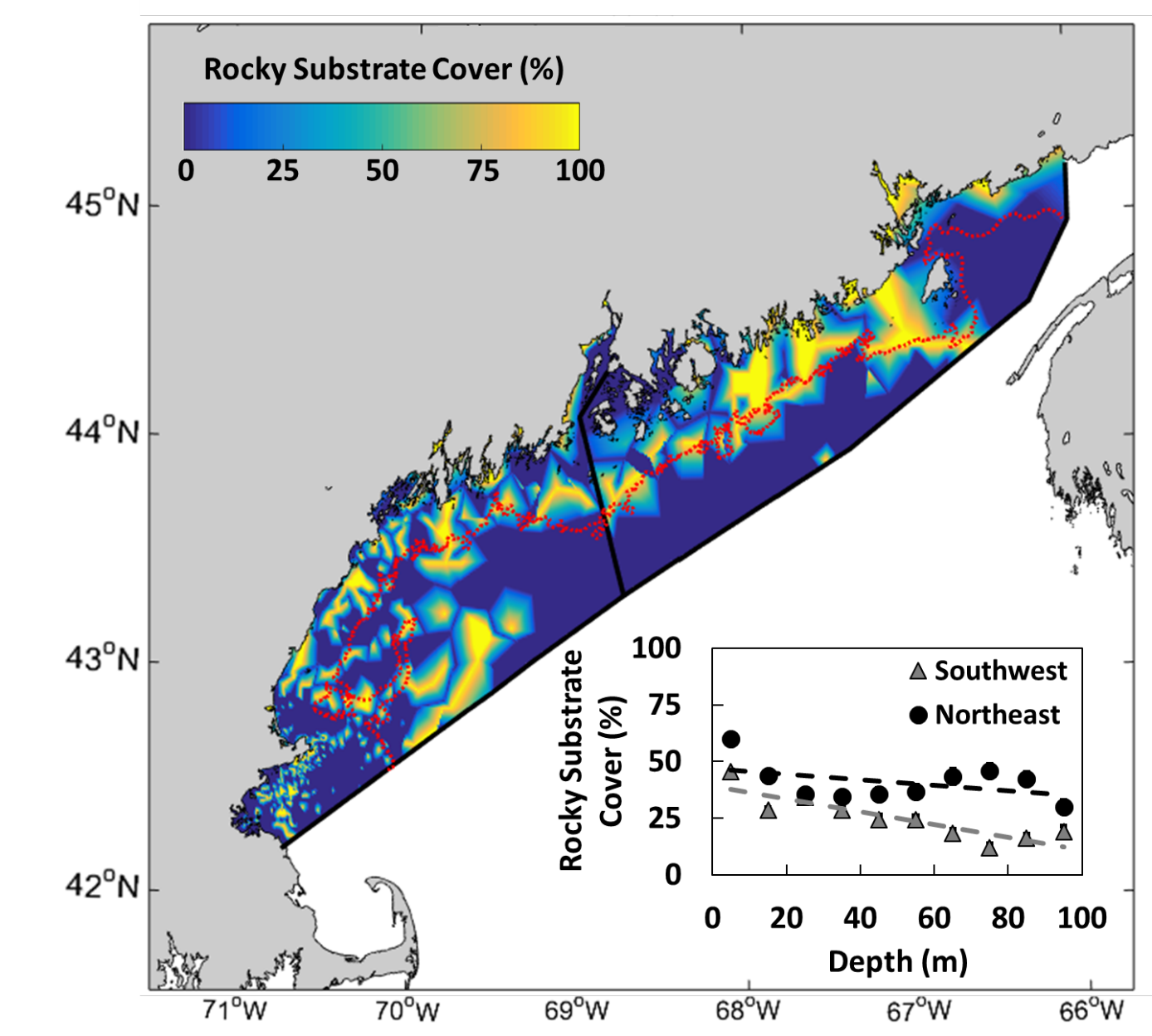


**Supplemental Figure 3**. Rocky nursery habitat availability. Red dotted line: 100 m isobath. Inset: Rocky substrate percentage cover < 100 m. Data points: regional average grid percentage rocky substrate cover per 10 m depth bin. Error bars: standard error. Dashed lines: least-squares linear regressions. Southwestern Gulf of Maine: Y = -0.28x + 39.26, N = 10, R^2^ = 0.77, p = 0.001. Northeastern Gulf of Maine: Y = -0.12x + 46.86, N = 10, R^2^ = 0.19, p = 0.204.


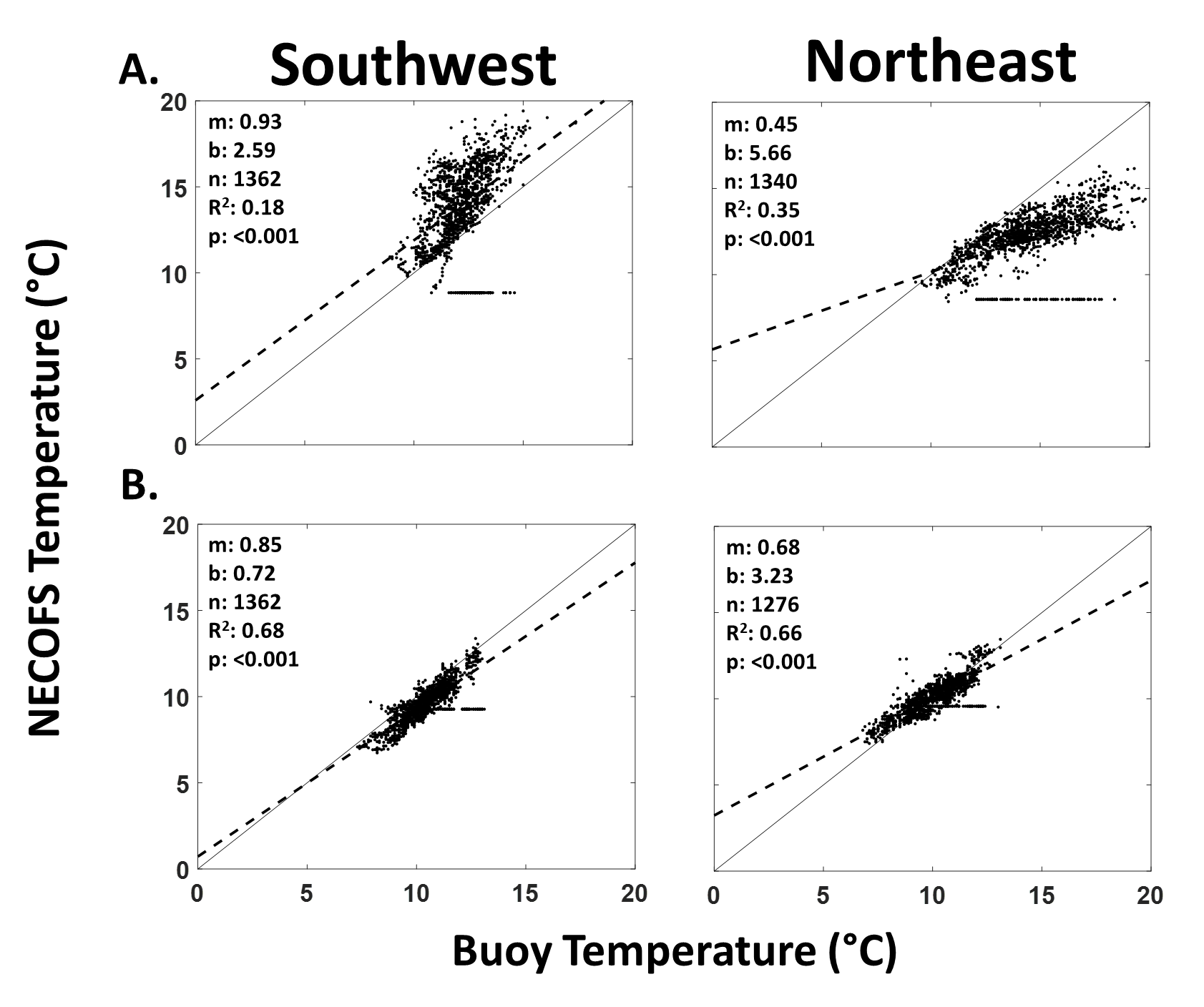


**Supplemental Figure 4**. Relationship between Aug-Oct. buoy (NERACOOS buoy E01 and I01) and modeled (NECOFS) temperatures from 2001-2015 at the A.) surface (1 m), and B.) bottom (50 m). Solid line denotes 1:1 relationship. Dashed line denotes least-squares linear regression. Inset: regression statistics.


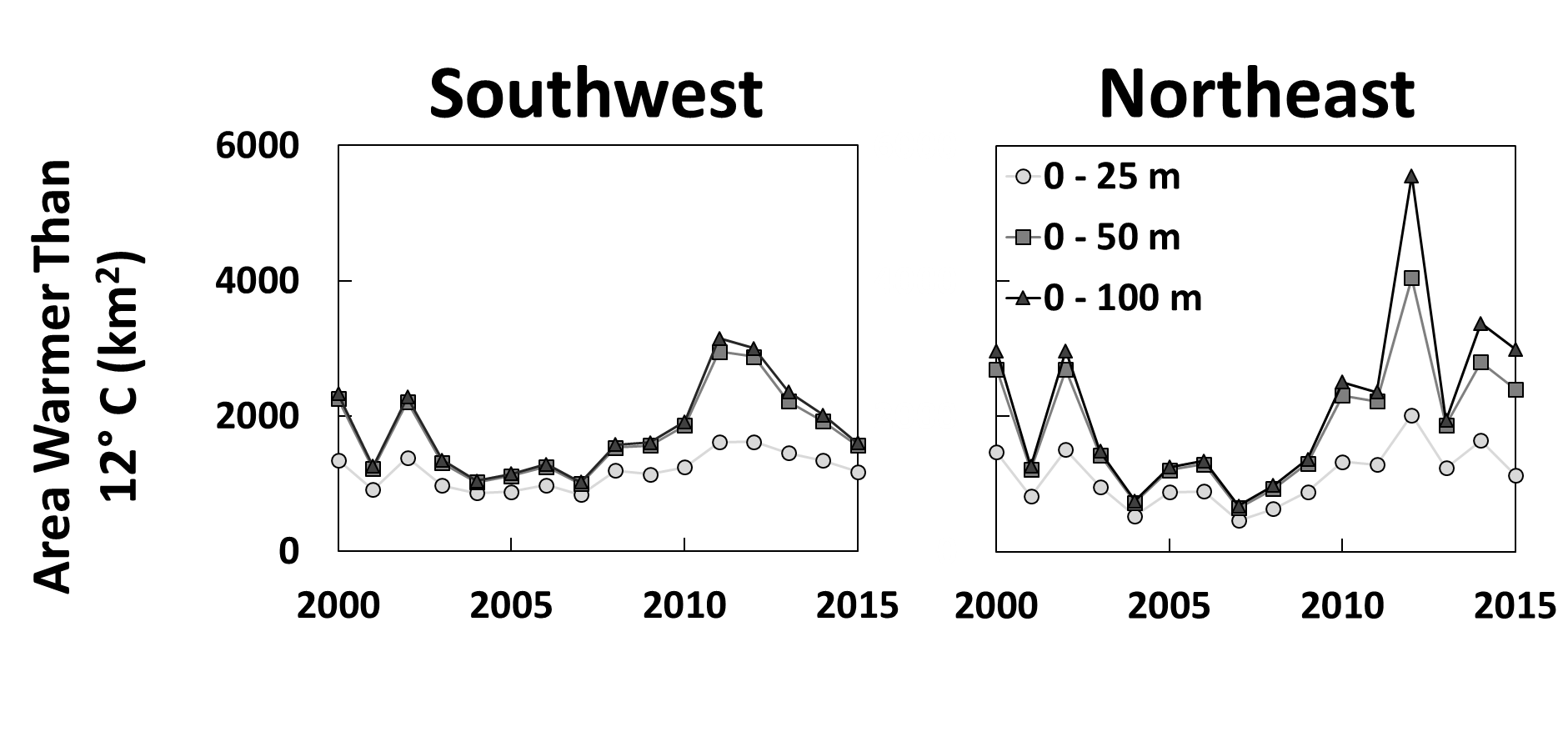


**Supplemental Figure 5**. NECOFS-modeled bottom habitat with temperatures >12 °C (km^2^) shallower than 25, 50, and 100 m in the southwestern and northeastern Gulf of Maine from 2000-2015.


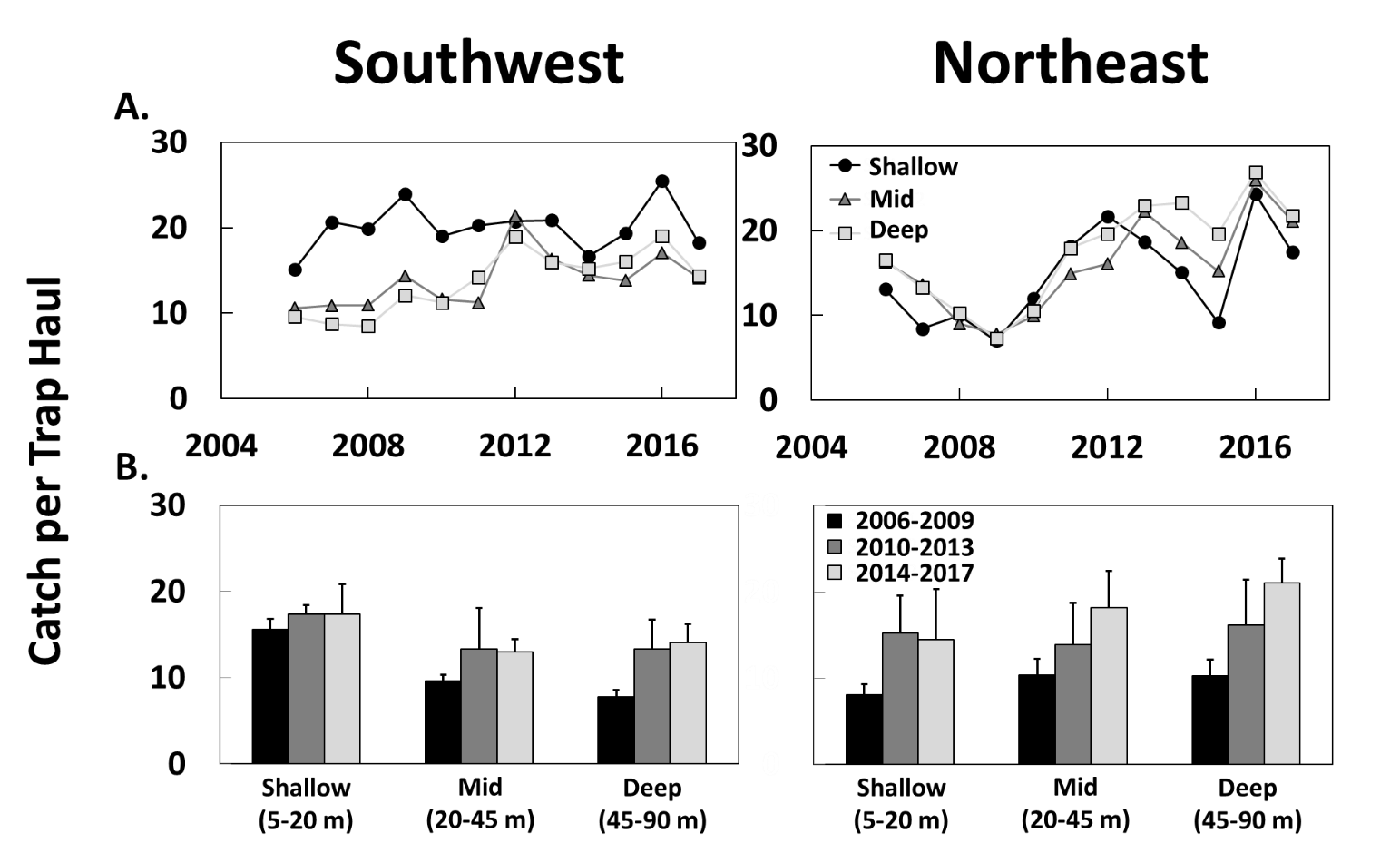


**Supplemental Figure 6**. Depth-specific catch per trap haul of 53-83 mm carapace length lobster in the Gulf of Maine from the Maine Department of Marine Resources ventless trap survey. A) catch time-series from 2006-2017. B) Four-year block average catch; values are average + standard error.

**Supplemental Table 1**. Least-squares regression summary statistics between various depth-limited thermal habitat areas and young of year lobster densities in the southwestern and northeastern Gulf of Maine.

**Supplemental Table 2**. Least-squares regression summary statistics between various temperature data and the American lobster settlement index.

**Supplemental Table 3**. Least-squares regression summary for Figure 2A. Values are aggregated slopes (°C year^-1^) for all NECOFS grids within each region and depth stratum. A post hoc Tukey HSD test showed significant differences in warming rate between region and depth stratum at the p < 0.05 level. Letters denote statistical similarity.
